# Facilitating laboratory automation using a robot with a simple and inexpensive camera detection system

**DOI:** 10.1038/s41598-025-05670-1

**Published:** 2025-06-20

**Authors:** Rebecca Wienbruch, Nicole Rupp, Ruven Dreischke, Verena Mayer, Isabell Fenske, Rebecca Bauer, Thole Zuchner

**Affiliations:** 1 Faculty for Life Sciences, Professorship for Bioanalytics and Laboratory Automation, Albstadt-Sigmaringen University, Anton-Günther-Str. 51, 72488 Sigmaringen, Germany; 2 jetzt GmbH, Bücklestr. 3, 78467 Constance, Germany; 3 Neustadt Weinstraße, Germany

**Keywords:** Robotic arm, Bioanalytical lab automation, Raspberry Pi, Digital display recognition, 3D reconstruction, CAD design, Image processing, Machine learning

## Abstract

Laboratory automation has transformed bioanalytical research, yet smaller research laboratories face challenges in adopting such technologies due to limited resources, time, and technical expertise, while already facing complex bioanalytical methods. To address these barriers, we developed a robotic-arm-based camera detection system featuring two software applications designed to simplify laboratory automation. Both applications use fiducial markers (Augmented Reality University of Cordoba (ArUco)), for object detection. The first application creates a 3D digital model of the robot’s environment using ArUco markers and a Python-based Open Computer Vision (OpenCV) simulated stereo vision setup, enabling automated computer-aided design (CAD) in FreeCAD. This facilitates safe and efficient robot arm navigation. The second application integrates a deep learning neural network for automated digital display recognition, achieving an in-house error rate of 1.69%, comparable to manual readings. By leveraging low-cost hardware and open-source software available on GitHub, the system is accessible to smaller research facilities, reducing programming complexity and enabling broader adoption of laboratory automation in bioanalytical workflows. This work demonstrates an affordable and effective solution for integrating robotic arms into scientific workflows, enhancing reproducibility and efficiency in bioanalytical research.

**Supplementary Information:**

The online version contains supplementary material available at 10.1038/s41598-025-05670-1.

## Introduction

The advantages and challenges of laboratory automation in the academic-scientific field have been widely discussed^1^. Laboratories and their processes are highly diverse, making it impossible to provide a universal recommendation for a specific automation system^2^. Bioanalytical laboratories often automate only specific processes—such as sample preparation, assay execution, and data analysis—temporarily, as academic researchers usually face time-limited contracts. This makes it impractical to replace existing equipment with expensive automated devices^1^. In these situations, integrating a robotic arm into the laboratory that can work with existing manual laboratory equipment and without extensive reprogramming would be highly beneficial.

However, automating existing laboratory instruments, such as pH meters or shakers presents challenges. Scientists in bioanalytical fields already manage complex and demanding workflows. Manually teaching the robotic arm precise positions can be tedious and requires skills outside their conventional expertise, significantly reducing the user-friendliness of these systems^3^. Two approaches could assist scientists in achieving smooth automation under these circumstances. First, virtual mapping of the robotic arm’s environment could help prevent collisions with obstacles such as laboratory devices, shelves and tables, enabling automated, collision-free path planning. An integrated computer-aided design (CAD) feature would enhance safety, increase adaptability to changes in device placement and reduce setup effort. Instead of requiring manual measurements and external CAD file generation, the system could automatically produce a usable CAD of the environment, allowing the robot to plan safe and efficient movement between the robotic arm’s current and target positions.

Secondly, many laboratory devices lack built-in interfaces, which complicates their integration into automated workflows. Automated display read-outs could significantly increase reproducibility, as manual data handling is error-prone. Incorrect data can influence critical bioanalytical workflow steps like liquid handling, incubation times, data analysis and consequently the experimental results. For clinical data, error rates from 0.5–8.8% and some variations being off by more than 20% are common^4–6^. While error rates vary, all studies highlight the risks for the patients and in particular, wrong results and their interpretation can have a negative influence on medication prescriptions in the medical field^7^. Digitally connected devices or automated display read-outs for non-connected laboratory equipment could help reduce these errors.

To address these challenges, we developed a camera-based recognition system that operates independently of device interfaces and positions to facilitate the use of robotic arms in scientific laboratories. The system is capable of accomplishing both tasks: reading digital values from laboratory device displays and generating virtual representations of the laboratory environment. These capabilities are demonstrated using a six-axis robotic arm equipped with an inexpensive Raspberry Pi camera system. Augmented Reality University of Cordoba (ArUco) markers serve as physical reference points to identify the display region for LCD read-out and to extract object dimensions for CAD-based visualization of the laboratory setup.

## Materials and methods

To identify the best methods for our camera detection system, we analyzed the state-of-the-art. Concerning the laboratory mapping, deep learning has become the state-of-the-art in this field, with various applications described in the literature^8–10^, like semi-automated CAD design methods for personalized medicine^11,12^. While deep learning is useful for high-accuracy tasks, large-scale datasets are necessary to increase the applicability of deep learning methods^13^. Our application requires only the outer dimensions of devices for collision prevention, allowing for simplified mapping using fiducial markers, and their known sizes. In comparison to point-cloud and deep learning techniques, a fiducial marker approach requires lower computational power and processing time.

To automate the readout of different liquid–crystal displays (LCDs), a deep learning (DL) approach is a valuable and state-of-the-art solution. Several publications describe neural network approaches for reading seven segment displays in the process control industry and approaches for medical monitoring. These methods use binary image information and statistical analyses to crop the images to the region of interest^7,14–16^. Standard optical character recognition (OCR) technologies, like Tesseract, were developed to recognize plain text with white background and black letters. In real-world conditions like reading text from images, noise, blurring, distortion and a variety of colours and fonts occur. Without specified pre-processing standard OCR techniques reach their limits^15^. Since standard laboratory devices typically derive from a wide range of manufacturers, their display technologies, colours, text, and digit formats vary significantly. In this case, a convolutional neural network (CNN) DL model presents a suitable solution. Artificial neural networks, such as CNNs, learn to identify and characterize complex data patterns by training on input data, which is then used to predict or classify new data. Additionally, it reduces the dimensions of an input image by decreasing the number of parameters and can be trained to handle the wide variety of laboratory displays effectively^17,18^.

**Fig. 1:**
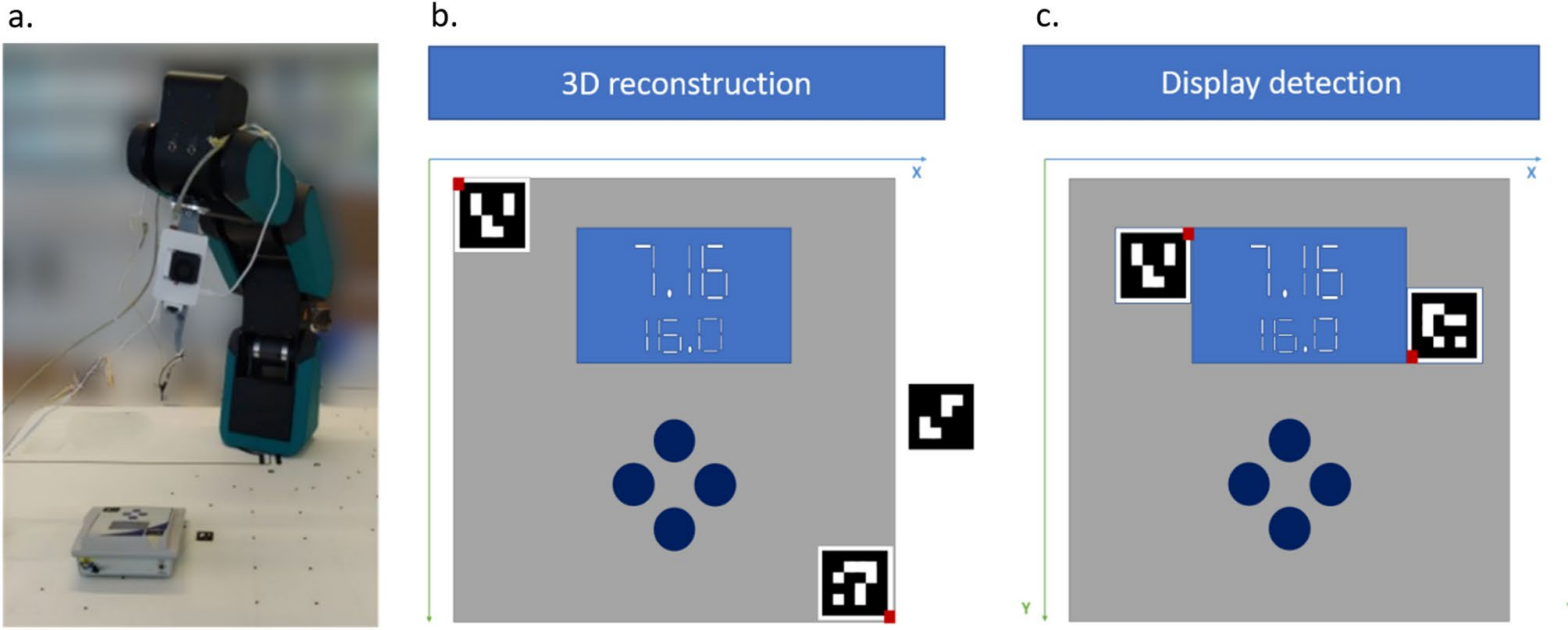
System overview using a pH meter as an example. (**a**) Camera integration with robotic arm: A camera mounted on the robotic arm moves within the workspace to detect markers. (**b**) 3D reconstruction: Three markers define the device dimensions. One marker on the surface beneath the laboratory device for height calculation; two markers on the device’s corners for width and length estimation. (**c**) Display detection: Two ArUco markers define the display area, aligned at the display’s top-left and bottom-right corners (red corners).

The detection system (Fig. 1a) is based on a Raspberry Pi Camera Module 2 (Raspberry Pi Trading, South Cambridgeshire, UK), connected to a Raspberry Pi 4 Model B (2018) board (Raspberry Pi Trading, South Cambridgeshire, UK). The information detected by the camera system (e.g., current camera image or current visible marker IDs) is displayed via a browser interface. The camera is mounted on the 6-axis robotic arm Horst600 (fruitcore robotics GmbH, Konstanz, Germany) using a 3D printed adapter to allow for automatic camera movement.

We programmed the robot using horstFX software version 2022.07 (fruitcore robotics GmbH, Konstanz, Germany) to create a scanning routine within the robot’s range of motion. AutoIt, a virtual Windows user scripting tool, enables the coordination of various Windows software through keyboard strokes and mouse clicks. In our system, an AutoIt script (AutoIt version 3.3.14.5, AutoIt Consulting Ltd., Worcestershire, UK) controls both the horstFX program and the camera interface. The script also provides a Graphical User Interface (GUI) that allows the user to choose between 3D reconstruction and display detection.

For both applications we used fiducial monochrome square ArUco markers for laboratory device identification and localisation, as their detection is considered robust, fast and simple^19^. Each marker encodes a specified ID between 0 and 999 in a unique 4 × 4-bit pattern, with a side length of 0.025 m, ensuring a fixed marker orientation relative to the camera. Figure 1 provides an overview of the setup, highlighting the exact placement of ArUco markers for 3D reconstruction and display detection. Even if the display is small relative to the marker, it still can be recognized. In case of 3D reconstruction (Fig. 1b), the user must position two markers at opposite corners of the device’s outer edges. A third ArUco marker has to be positioned on the surface the device rests on to capture its height. The system captures two images (“image1.jpg” and “image2.jpg”) of the desired object (taken at a horizontal distance of 5 cm) and the corresponding coordinates in a text file format (“pos1.txt” and “pos2.txt”). For successful display detection, the user must position the two markers as shown in Fig. 1c: one marker at the upper left corner of the display and the other at the lower right corner. The system captures a single image with the corresponding coordinates in text files. Image processing is handled by Python scripts executed via AutoIt.

Python version 3.11.2 is used for digit recognition and Python version 3.8.1 for 3D reconstruction. The OpenCV library (version 4.6.0.66) was employed for ArUco marker detection. The function “detectMarkers()” identifies the marker IDs and their corner coordinates in the image’s pixel coordinate system, with the origin in the top-left corner of the image. The positive x-axis extends to the right, and the positive y-axis extends downward. Since the orientation of ArUco markers affects further data handling, we implemented a sorting algorithm to avoid conflicts due to rotated ArUco markers^19^. The corner coordinates of each marker are stored in a consistent order: top-left, top-right, bottom-right, and bottom-left, regardless of the marker’s rotation in the image frame. The system automatically detects and arranges the marker orientation by rearranging the marker’s x- and y-values. This ensures the correct identification of marker positions in the image^19^.

For laboratory mapping, the corner pixel coordinates and marker IDs are used to calculate the device dimensions (length, height and depth) through 3D reconstruction, translating the pixel coordinates to the robotic arm’s world coordinate system. For display detection, we cropped the image to display size, based on the marker corner coordinates to reduce the noise ratio.

### Laboratory mapping

Laboratory mapping was performed by transforming 2D marker pixel coordinates into a 3D representation with Python. The CAD models are generated directly from 2D marker pixel coordinates in FreeCAD (version 0.21.1, freecad.org), a community-based, open-source CAD software compatible with Python. Transforming coordinates requires the camera’s intrinsic (lens distortion and focal length) and extrinsic (rotation and translation vectors) parameters. Extrinsic parameters, define the camera’s pose relative to the reference coordinate system ensuring accurate 3D reconstruction^20,21^. The extrinsic parameters for each position are recorded in a text file generated by HORSTFX via AutoIt3.

The intrinsic camera matrix and distortion coefficients were obtained through geometric camera calibration^20,22^, using Python (version 3.12.0), OpenCV’s “calibrateCameraCharuco” function (OpenCV version: “opencv-contrib-python 4.8.1.78”), the NumPy (Numerical Python) library (“numpy 1.26.2”) and a 2D ChArUco (Chessboard Augmented Reality University of Cordoba) board, created on “www.calib.io”, attached to a planar surface. The 210 × 297 mm board included 18 black chessboard squares (side length: 34 mm = 0.034 m) and 17 ArUco markers (side length: 25 mm = 0.025 m) from the 4 × 4 ArUco dictionary. Using the Raspberry Pi version 2 camera module, 165 images were captured from various angles and distances for calibration. The resulting intrinsic matrix and distortion coefficients^20^ were used to undistort images (cv2.undistort()). To reconstruct the 3D marker positions, we employed triangulation. Rather than a stereo camera setup, we simulated stereo vision by capturing images from two different camera positions. The intersection of these perspectives provides depth information^23^.

### Display detection

The training datasets were produced with Roboflow (https://roboflow.com/), a platform for computer vision applications, where objects in images (e.g.‘.’, ‘pH’, ‘temp’, ‘°C’, ‘min’, 0–9) were manually labeled^24^. Model training was done on Kaggle (https://www.kaggle.com/), using the GPU acceleration (only available after account verification) and a Python code incorporating the Ultralytics library and the Roboflow dataset in yaml format. Images were resized to 640 × 640 pixels and training lasted up to 300 epochs or until convergence. The best performing model was saved as “best.pt”. The use of such third-party applications like Roboflow and Kaggle require public data availability, hence no sensible data should be uploaded for training, to prevent data privacy concerns, or produce training datasets and trained models locally.

Each display is identified by its marker ID pair, linking it to predefined display characteristics (e.g. values limits and decimal places. The cropped image is processed by the trained CNN model, which returns bounding boxes, confidence intervals, and class labels for each digit. A Python script sorts predictions left-to-right by x-coordinates and, for multi-row displays, splits data frames based on bottom y-coordinate differences (> 0.8 pixels).

Overlapping predictions are flagged if the bottom x-coordinate differences are between -0.002 and 0.002 pixels, and duplicates are resolved by keeping the prediction with the highest confidence value. Large gaps (> 0.2 pixels) between bounding boxes x-bottom coordinates are used to identify separate values, on single row displays. The workflow also corrects decimal placement, replaces non-numeric labels such as “OFF” with numerical equivalents (e.g., “0”), and validates outputs against expected ranges. Final results (float values, confidence intervals and marker IDs) are stored in an “output.csv” file and further processed into a structured Excel file, ensuring user-friendly and traceable results.

## Results & discussion

The developed camera detection system enhances the automation of robotic arm tasks, especially for academic bioanalytical research laboratories. The system, along with setup scripts (e.g. camera calibration script), is available as open source on GitHub*.*

### User-friendliness

**Fig. 2:**
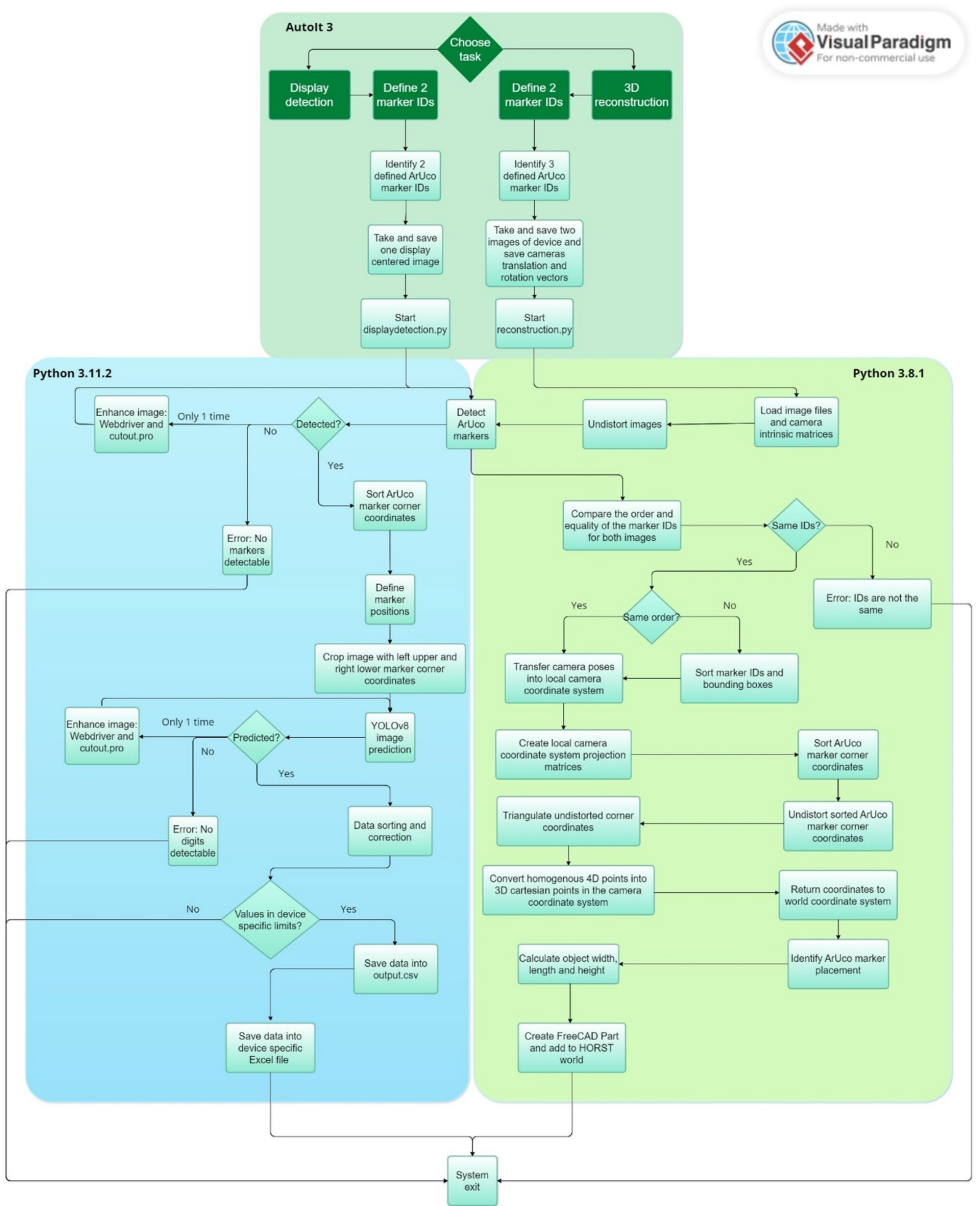
Process flow of the camera detection system. The AutoIt script guides the user through a simple interface, mainly using mouse clicks (user steps in dark green). After choosing between display detection or 3D reconstruction and specifying ArUco marker IDs, the corresponding Python process runs automatically.

Figure 2 outlines the process flow of the camera detection system. The user interaction for both tasks—automating the creation of a CAD environment and display recognition—is handled through a single AutoIt script with a simple GUI, enhancing the user-friendliness of the system for bioanalytical scientists. In this GUI, the user selects the task (either display recognition or 3D reconstruction) and inputs the required ArUco marker IDs (two or three) by clicking on the desired options.

While Python and AutoIt must be installed manually once in the beginning, we automated the setup of the Python virtual environments and package installations to simplify this complicated and error-prone process, enabling even occupied bioanalytical scientists to implement the system easily. After running the “Python_installation.au3” AutoIt script, the user only has to define the user-specific paths, as described in Supplementary 1 and in Supplementary 1, Table S1. After that everything is ready for use. However, if more Python packages or virtual environments are needed, instructions are provided in Supplementary 1, and these tasks can also be managed by a system administrator. Additionally, all Python packages used are listed with the version numbers in Supplementary 1, Table S2, S3 and S4.

Once the system is running, the display detection module generates an Excel sheet containing relevant data, while the 3D reconstruction module creates a STandard for the Exchange of Product model data (STEP) file. This file, a format commonly used in CAD for 3D models, provides a simplified representation of the obstacle of interest. Any further use of the Excel and STEP files is the user’s responsibility, as subsequent processing depends on the specific system and task requirements. Handling these files is straightforward for any user, requiring only basic interactions such as mouse clicks and keyboard inputs to manipulate the data or upload the STEP file into the robot’s software.

To implement the system into a workflow with another robotic arm, some adjustments would have to be made in the AutoIt script. We provide the AutoIt script for the horstFX implementation, which can be used as an orientation. Due to the comments in this AutoIt script, the logic can be easily adapted to other robotic arm software.

### Automated CAD design—3D reconstruction

**Fig. 3:**
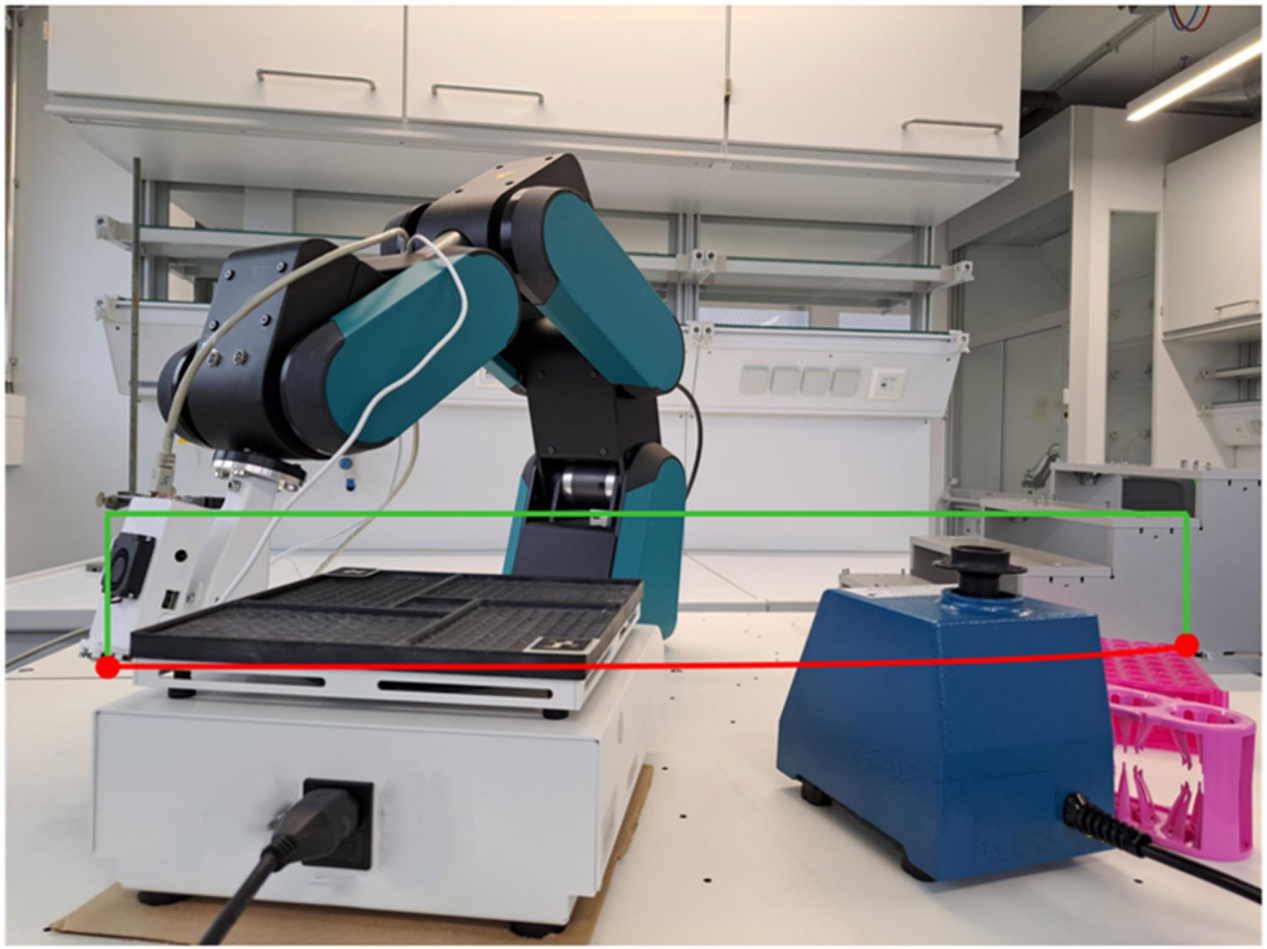
Visualization of robotic arm pathways and device 3D reconstruction. Robotic arm movement pathways within its workspace: The red line represents the shortest direct pathway; the green line reflects the adjusted pathway after integrating 3D reconstructed lab devices.

Figure 3, illustrates how the environment influences robotic path planning. Instead of following the shortest direct route (red line), which would result in a collision with obstacles, the robot software calculates and adjusts the trajectory (green line), when a CAD environment is provided, to ensure safe operation. This highlights the benefit of incorporating environmental context into robotic control for greater flexibility and safety. To make this process easier, we developed an automated 3D reconstruction system that creates this virtual environment with minimal user input.

The system sorts and corrects (undistorts) marker positions to increase position accuracy, then triangulation transfers the 2D image coordinates into a 3D coordinate system, adding depth information (detailed information in Supplementary 2). After the 3D coordinates are determined, they are aligned with the robot’s coordinate system for accurate CAD design and movement planning.

**Fig. 4:**
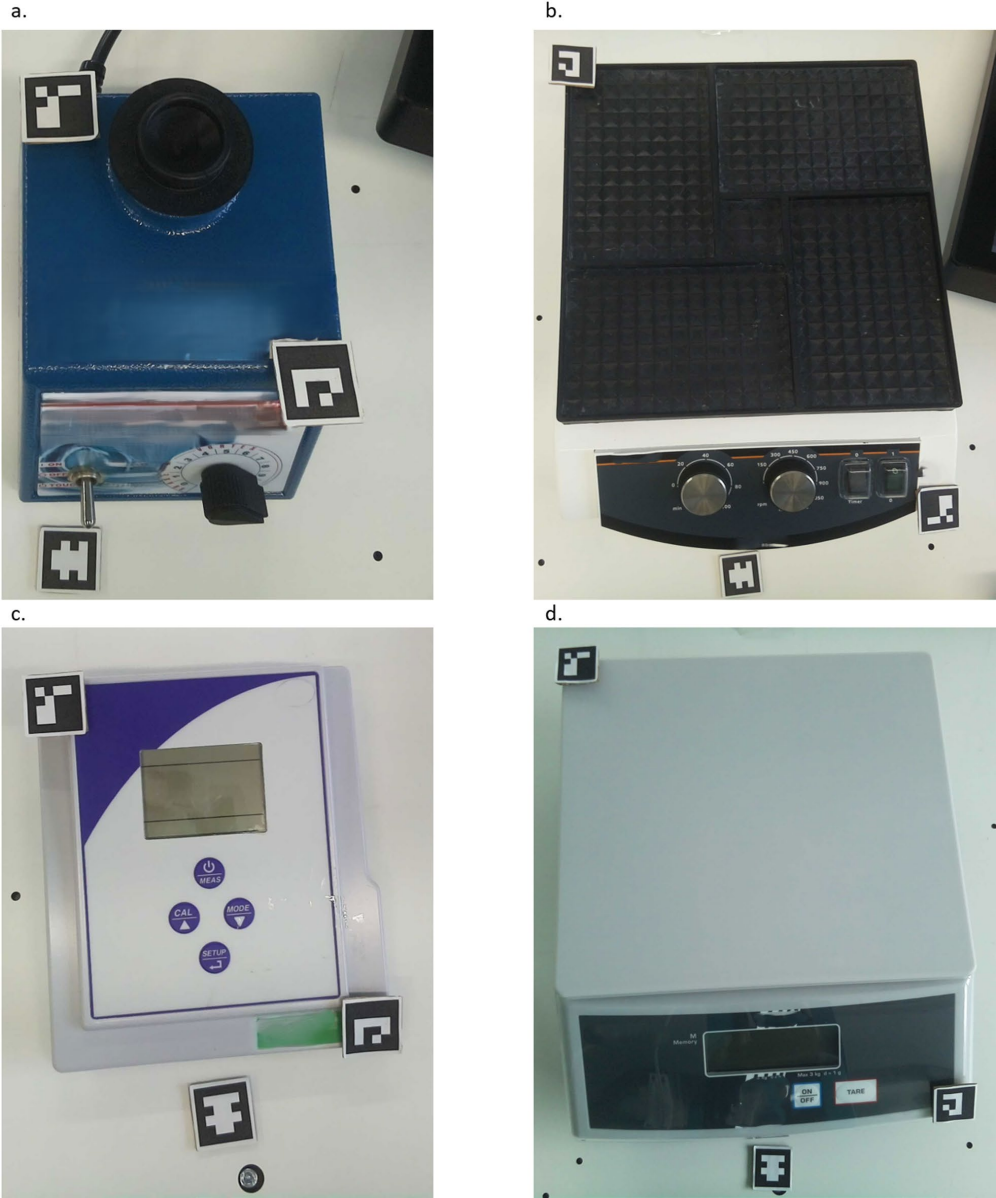
Reconstruction measurement devices. (**a**) Vortexer (**b**) Plate shaker (**c**) pH meter (**d**) Scale.

For quantification of the 3d reconstruction accuracy, ground truth measurements were compared to 3D reconstructed marker distance measurements. Additionally, the measurements were compared to the dimensions provided by the manufacturers. In total four devices, shown in Fig. 4 were used for this quantification. The devices vary in their sizes, as well as the surface angles.

The quantification results are shown in Table 1. Mean deviations were 12 mm in the X-direction, 10 mm in the Y-direction, and 23 mm in the Z-direction. Despite the presence of angled surfaces, the reconstruction of marker distances was generally successful, demonstrating reliable 3D measurement performance across all tested devices. The largest errors occurred with the pH meter, where its sloped geometry introduced greater inaccuracy during reconstruction. Notably, Z-direction deviations were highest, as accurate height determination requires a perpendicular (90°) viewing angle onto the markers. Across all devices, individual error values ranged from 2 to 34 mm.

**Table 1:** Reconstruction result comparison to ground truth measurements in mm.

	Vortexer	Plate shaker	pH meter	Scale
Marker X	114	235	161	265
Marker Y	129	320	175	310
Marker Z	122	130	62.44	113
Reconstruction X	120	255	181	263
Reconstruction Y	125	301	180	298
Reconstruction Z	150	111	28	123
Diff. Marker X	6	20	20	− 2
Diff. Marker Y	− 4	− 19	5	− 12
Diff. Marker Z	28	− 19	− 34	10

The reconstructed width (X), length (Y) and height (Z) values are compared to the manual marker distance measurements. Even for devices with angled surfaces, the overall mean deviation of 15 mm demonstrates a high level of accuracy and remains well within acceptable tolerance limits. As the software automatically adds a safety margin of 50 mm in every dimension, no collisions can occur.

To enhance safety, we added a 5 cm buffer to each device’s dimensions to prevent accidental collisions. This buffer accounts for variations in calculated marker distances, which may deviate by several millimetres in all three dimensions. More importantly this buffer minimizes the error for marker placement problems. Optimizing the angle between the camera and markers (ideally 90°) minimizes marker distance inaccuracies by reducing perspective distortion.

Overall this 3D reconstruction system provides a fast and accessible way to add new devices to the robot’s CAD environment, improving safety and flexibility without requiring CAD expertise. While the CAD model must be uploaded and integrated manually, depending on the robot software platform, our system allows scientists without CAD experience to set up a virtual laboratory environment for robot applications. Although CAD model import is supported in many robot software platforms, it is not a universally standard feature, and some systems—such as horstFX—do not automatically treat imported CAD models as physical obstacles for simulation or collision detection. This makes setup more complex as users have to manually adjust the paths using visual inspection. However, this tool represents a valuable advancement as robotics continue to evolve.

### Display detection system

To achieve accurate display detection, we trained ten sequential YOLOv8 (You Only Look Once version 8) CNN models to identify seven-segment digits from various laboratory displays. Each iteration is building upon and improving the previous one. YOLOv8, available on Ultralytics (https://docs.ultralytics.com), is a fast, Python-compatible CNN for real-time object detection and image segmentation, ideal for our display detection workflow^24,25^.Training data included images from six devices, commonly used in bioanalytical workflows: a pH meter, a heating and stirring plate, a centrifuge, two scales, and a thermo-shaking drybath (with eight-segment digits). These devices represent diverse formats, colours, and layouts.

**Fig. 5:**
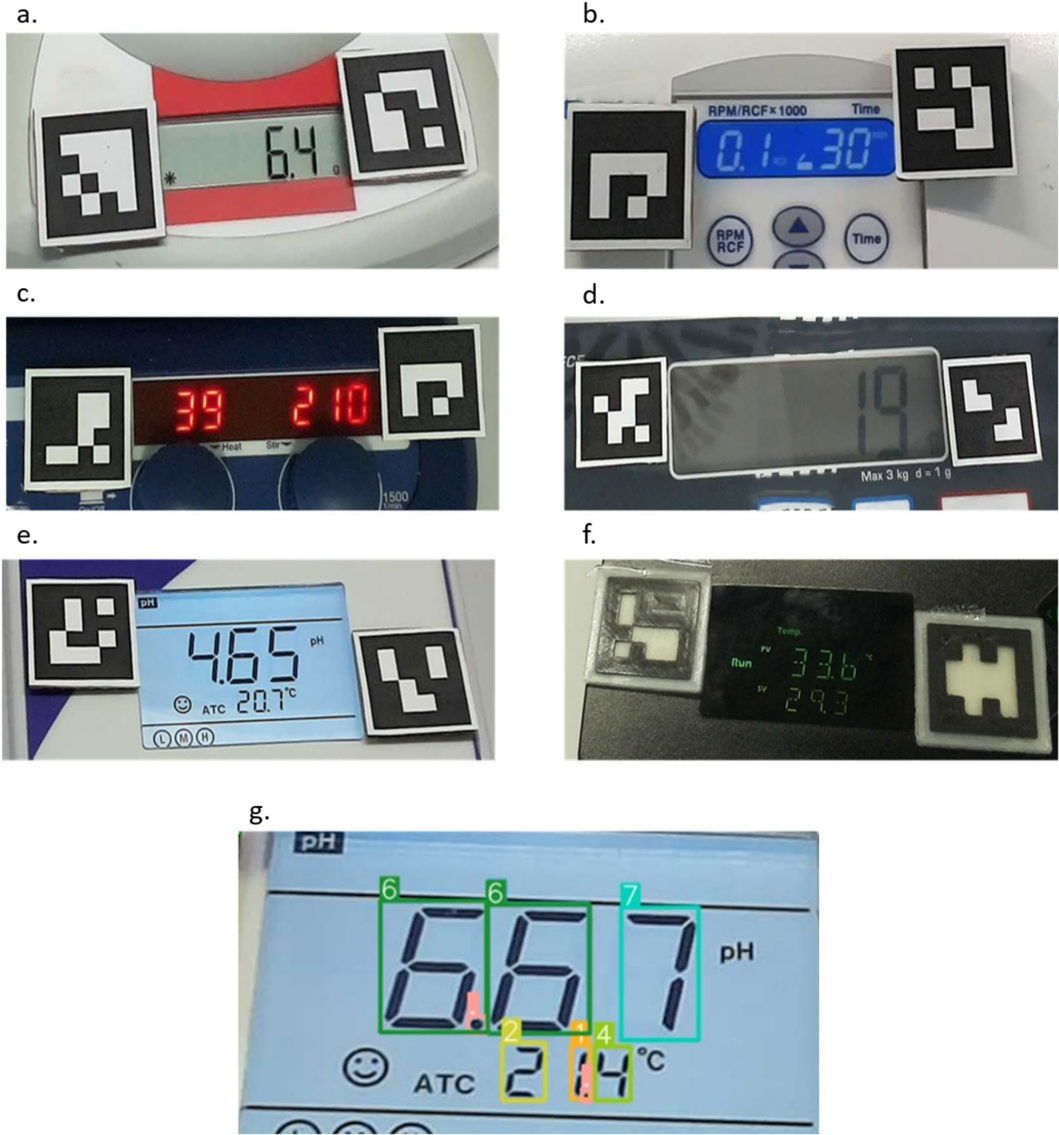
Trained laboratory displays. (**a**) Scale single value, one decimal place. (**b**) Centrifuge: two values, left value one decimal place, one row. (**c**) Heating and magnetic stirring plate: two values, no decimal places, one row. (**d**) Scale: one value, no decimal places. (**e**) pH meter: two values, two decimal places upper value, one decimal place lower value, two rows. (**f**) Digital Shaking Drybath: two values, one decimal place each, two rows, using eight-segment digits. (**g**) Trained model prediction result for display **e.** showing accurate detection of all digits. Coloured boxes indicate predicted digits; labels above boxes show class names.

Figure 5 shows the various types of displays trained in our laboratory. Displays a-d represent values in a single row, while displays e and f represent values in two rows. The digits in displays a and d are black and set against a grey background. Unlike the other four displays, these lack integrated lighting. All displays, except for display f, use seven-segment digits to represent values. Display f, in contrast, uses eight-segment digits. In addition to the digits “0” to “9” and the “.” symbol, all displays except for c include additional symbols. The digit and background colours vary across all displays. Additionally, Fig. 5g shows the prediction results for the pH meter, which displays pH values (upper row, two decimal places) and temperature (lower row, one decimal place). The coloured bounding boxes indicate the areas where the model detected digits, with small numbers representing the detected classes.

#### Model training and evaluation

Precise image recognition and interpretation require extensive training of neural network-based models. Initially, we used publicly available image datasets (472 images from “shrinand aggarwal”^26^ and 390 images from “diego”^27^), sourced from Roboflow. However, the image quality of these external datasets proved insufficient for effective augmentation. Consequently, all subsequent models were trained exclusively on laboratory-acquired images.

For model evaluation, the so called mAP50 (mean Average Precision for an IoU (Intersection over Union) threshold of 50%) parameter was used, which measures detection accuracy^25^. A more detailed description of the mAP50 value is provided in Supplementary 2. Additionally, all trained model’s mAP50 values are shown in Supplementary 2, Fig. S1. We divided the datasets into subsets: a training set (70–90%) and a validation set (10–30%). For testing we used 10 images of each display.

**Fig. 6:**
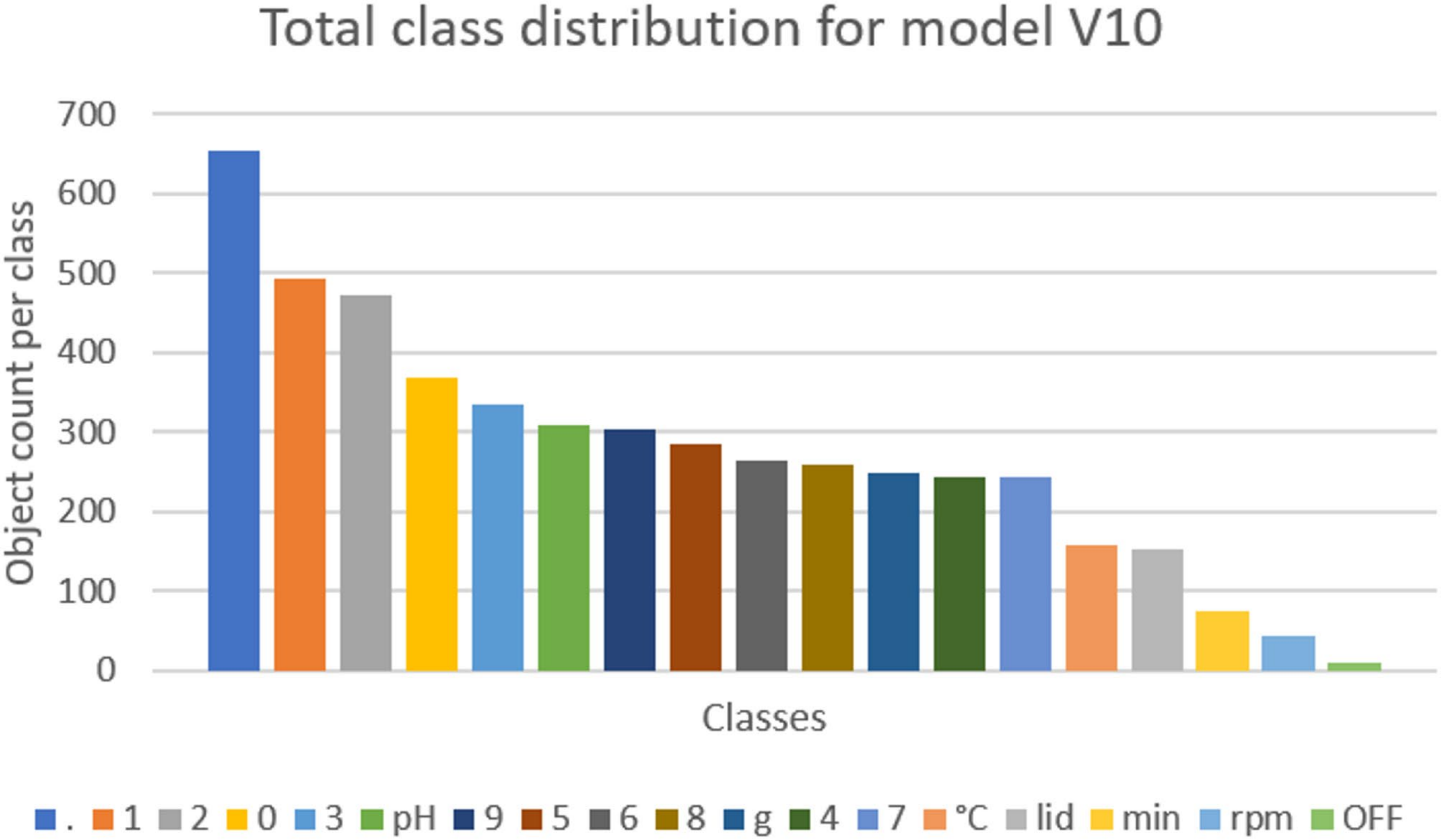
Total class distribution for trained model V10. Most classes are nearly homogenic. Class “.” is nearly in every image. The classes “°C”, “lid”, “min”, “rpm” and “OFF” are only present on a single device type.

Finally, the last two trained models (V9 and V10) showed promising results. While model V9 (mAP50 = 0.969) achieved relatively high prediction rates, the refined model V10 (mAP50 = 0.956) demonstrated even better performance. Model V10 was trained with 2241 images and validated with 66 images, using three augmentations (exposure + /− 10%, blur up to 0.8 px and noise up to 1.88% of pixels) and included 18 classes (numbers and symbols). Its superior performance can be attributed to the exclusion of the eight-segment display (Fig. 5f) and a more homogenized display image distribution (Fig. 6). A detailed class distribution for model V10 is shown in Supplementary 2, Table S1. Model V10 achieved prediction rates of 90% or higher for all five tested devices (Table 1).

Table 2 summarizes the display characteristics, value ranges and prediction results for each included device. Displays with simpler numeric formats, such as those without decimal points, showed higher prediction accuracy. Performance was further enhanced by a Python-based correction script, which successfully addressed missing decimal points.

**Table 2:** Summary of the tested laboratory displays.

	Scale	Centrifuge	Scale	Heating and stirring plate	pH meter
Font	Black	Light blue	Black	Red	Black
Background	Grey	Dark blue	Light grey	Dark blue	Light blue
Illumination	No	Yes	No	Yes	Yes
Value 1 limits	0–3000	1.2–13.5	0.0–200.0	5–280	0–14
Value 2 limits	–	1–30	–	200–1500	0–100.0
Value 1 decimal places	None	1	1	None	2
Value 2 decimal places	–	None	–	None	1
Tested images	10	10	10	10	10
Value 1 correct	100%	100%	90%	100%	90%
Value 2 correct	–	90%	–	100%	100%
Sum detectable digits	19	44	40	54	80
Sum correct digits	19	43	39	54	80
Sum false digits	0	1	1	0	0
Sum missing digits	0	0	0	0	0
Sum additional digits	0	0	0	0	2
Sum detected digits	19	44	40	54	82
Error rate	0.00	0.02	0.03	0.00	0.03
Recall	1.00	0.98	0.98	1.00	1.00
Precision	1.00	0.98	0.98	1.00	0.98

The display detection using model “V10” resulted in 90 to 100% correctly detected values across all five devices.

#### Influence of image enhancement and lighting conditions

Lighting conditions and image quality significantly affected digit recognition. Poor lighting reduced contrast between ArUco markers and their background, while excessive reflections hindered marker detection. For these situations we integrated a web driver-based Python script, that activates the third-party AI-based image enhancement tool from cutout.pro (https://www.cutout.pro/photo-enhancer-sharpener-upscaler), which is used when the marker or digit recognition failed. The image enhancement is limited to simple contrast and image quality enhancement, as only a generalized enhancement is applicable for the broad display varieties. The use of an online tool requires more time for the detection, a network connection and may present a data privacy concern as the image has to be send to a third-party server. Hence, the implementation of an offline alternative (like Real-ESRGAN) could be advisable where this is of concern.

To evaluate the benefit of an additional image enhancement, we took 20 images under four lighting conditions (from nearly no lighting to all lights turned on) from each of the five predictable laboratory displays (= 100 images). Automated image enhancement improved marker detection in 22 images, resulting in 87 predicted images, compared to 78 without automated enhancement. However, extreme lighting conditions (too dark or overly bright) or poor camera placement could still impede predictions (13 images). The need for image enhancement was limited and can be reduced to a minimum by ensuring good lighting conditions.

For example, the pH meter’s illuminated display occasionally caused overexposure, making digits unreadable even manually. Overall, ensuring appropriate lighting, optimal camera distance, and marker placement is critical for accuracy. If lighting conditions in the laboratory are rather bad or the camera’s distance to the display is too short, an automated image enhancement can be beneficial. A higher-quality camera with autofocus could further improve results.

#### Data handling, error analysis and evaluation

We compared our detection system to a standard OCR Tesseract system for further validation. Testing under controlled lighting conditions (all laboratory lights on) yielded 50 images with 237 predictable digits across five devices. The detailed results for the unrefined OCR Tesseract system are shown in Supplementary 2, Table S2. Without fine-tuning the Tesseract OCR model, it only detected 82 digits in total, with 44 correct detections. The error rate, including missing, false, and additional digits, is roughly 83%. This reflects the limited suitability of the unrefined Tesseract OCR system for reliably detecting LCD displays. The low recall (0.19) suggests that most relevant digits are missed, while the moderate precision (0.54) indicates that many of the detected digits are incorrect or irrelevant. For accurate results a fine-tuning of the Tesseract v4 LSTM model would be necessary. Like for our detection system, a large training data set would be necessary to fulfill the task.

Looking at the results of our display detection system (Table 2) with the trained YOLOv8 model, 235 out of 237 digits were correctly identified, with two errors (digit confusion of “2” with “5” and “0” with “8”) and two additional detections. Python-based data processing ensured that values outside device-specific limits were flagged, halting further data processing. We therefore conclude that the total in-house error rate of the system is roughly 1.69% which is comparable to or even better than manual data handling error rates described in the literature^4–6^. The system achieved a low error with a high recall of 0.99 and precision of 0.98, indicating both highly accurate and nearly complete detection performance. This error rate is an in-house value, for applying the system with our own devices. If other LCD display formats with significant variations to our trained displays are used, the error rate could be higher. The larger the training dataset will become in the future, the better the system will recognize variations of the displays analyzed. Nevertheless, the system’s performance underlines its reliability and potential for further optimization through expanded training datasets and improved hardware.

## Conclusion

This work introduces a cost-effective, user-friendly camera detection system designed to simplify the integration of stand-alone laboratory devices into automated bioanalytical workflows. By enabling the automated read-out of digital displays and the creation of a virtual lab environment in a CAD model, our system addresses key challenges in laboratory automation, making robotic arms more accessible and practical for use in academic research.

The system reduces the barriers for adopting automation in laboratories, requiring minimal technical expertise for setup. Researchers can install the software using straightforward instructions (Supplementary 2), and operate the system with basic interactions, such as mouse clicks and keyboard inputs. This ensures accessibility for a wide range of users, including those without extensive computational backgrounds.

The broader impact of this system is its potential to make automation more achievable for academic bioanalytical laboratories. By utilizing low-cost components and open-source software, it provides a scalable solution for research groups with limited budgets. Moreover, its modular design allows for customization and improvement by the scientific community, such as expanding training datasets, adding new display types, or adapting the system to different robotic platforms and cameras.

In conclusion, this project demonstrates a practical step forward in incorporating robotic arms into academic bioanalytical laboratories, making automation more accessible, reducing errors, and supporting more efficient and accurate experimental workflows. While not a complete transformation of laboratory practices, we hope that the system represents a valuable tool for advancing laboratory automation and fostering innovation in academic bioanalytical research.

## Electronic supplementary material

Below is the link to the electronic supplementary material.

Supplementary Material 1

## Data Availability

The data that supports the findings of this study are available in the Supplementary material of this article and the software is openly available in the GitHub repository “Lab_Auto_Camera_Detection” at https://github.com/LabAutoSig.
