# Supplementary material for: Facilitating laboratory automation using a robot with a simple and inexpensive camera detection system
Source: Sci Rep. 2025 Jun 20;15:20169. doi: 10.1038/s41598-025-05670-1 (PMC12181327; doi:10.1038/s41598-025-05670-1)
Supplement: Supplementary file 1 — Supplementary Material 1 [file 41598_2025_5670_MOESM1_ESM.pdf]

***Supplementary Information S1 for the Article:***

# Facilitating Laboratory Automation Using A Robot With A Simple And Inexpensive Camera Detection System

Rebecca Wienbruch[a] wienbruch@hs-albsig.de ORCID: 0009-0006-6649-9280, Nicole Rupp[a] rupp@hs-albsig.de ORCID: 0000-0002-7967-1346, Ruven Dreischke[b,1] ruven@dreischke.de, Verena Mayer[a] mayer_verena99@web.de ORCID: 0009-0003-6089-9134, Isabell Fenske[a] isabell.fenske@gmx.de, Rebecca Bauer[a] bauerreb@web.de ORCID: 0009-0000-0116-6908, Thole Zuchner[a,*] ORCID: 0000-0001-6095-4656

a: Faculty for Life Sciences, Professorship for Bioanalytics and Laboratory automation, Albstadt-Sigmaringen University, Anton-Günther-Str. 51, D-72488 Sigmaringen, Germany; b: jetzt GmbH, Schiffstr. 46, D-78467 Konstanz, Germany; [1]Am Speyerbach 44, D-67433 Neustadt Weinstraße, Germany

*<u>*Corresponding author:*</u>
Prof. Dr. Thole Zuchner, Email: zuechner@hs-albsig.de, phone: +49 (0)7571 732 8240

## 1 Detection system installation guide

The usage of the detection system requires installing AutoIt, FreeCAD, and multiple Python versions, along with their respective libraries. To manage different Python library versions on the same computer, virtual environments are necessary. This section provides a guide for installing AutoIt, FreeCAD and Python in virtual environments on a Windows computer. If you choose not to use the automated Python installation script we provided (AutoIt script) or wish to install additional Python versions, a detailed guide is included below. Additionally, two configuration files must be updated with your installation paths.

### 1.1 AutoIt 3 installation and interface

The AutoIt 3 package can be downloaded from the official website (AutoIt Downloads - AutoIt (autoitscript.com)). You can either download the executable "AutoIt Full Installation" and the AutoIt Script Editor (SciTe) installers, or download the AutoIt self-extracting archive (zip folder), if you lack administrative rights. After installation, type "SciTe" into the Windows search bar to open the editor. SciTe allows you to open existing AutoIt scripts or create new ones.

### 1.2 Python installation guide

To install Python, download the desired version's executable installer from the official Python website. For example, laboratory mapping requires Python 3.8.1, which can be downloaded from the Python 3.8.1 release page. Locate the "Files" section at the bottom of the page to find the executable installer. After download, first, start the installer and check the "Add to Path" option and secondly click "Install

now" to complete the installation. This process must be repeated for Python 3.11.2 and Python 3.12.0, which are required for display detection and camera calibration, respectively.

## 1.3 Virtual environments

If you use multiple Python version with version-specific libraries, create virtual environments. This is already the case if you want to use our display recognition and 3D reconstruction system on the same computer.

### 1.3.1 Automated Python installation script

We provide an automated AutoIt script for virtual environment and Python package installation, which you can use as follows:

1. Install AutoIt (see chapter 1.1)
2. Download the necessary Python versions (see chapter 1.2)
3. Double-click the "Python_installation.au3" script in the "camera_detection_system\Python_installation\" file
4. Wait until a message box is displayed that states that the last virtual environment is installed

### 1.3.2 Manual virtual environment and package installations

If you want to install other Python versions or additional libraries we provide a detailed guide below. The guide is written for the laboratory mapping Python version, but can be adjusted for any other Python version.

1. Python installation (chapter 1.2)
2. Open Windows command window with [Windows]+[R], type "cmd", and press ENTER.
3. Run the following commands:
    a. Write "py -3.8 -m pip install virtualenv" (replace "3.8" with your Python version) and press ENTER.
    b. Write "py -3.8 -m virtualenv venv38" to create a virtual environment named "venv38" and press ENTER.
4. To activate the virtual environment, type: venv38\Scripts\activate and press ENTER.
5. Once activated, install libraries using: py -m pip install <library name> == <version> and press ENTER.

6. Deactivate the environment by typing: venv38\Scripts\deactivate and press ENTER.

If you want to create the virtual environment in another path or access another virtual environment path, write “cd” followed by the desired system path and press ENTER. Afterwards, the current location is changed to the new path and you can type in the commands for virtual environment creation or library installation. The necessary libraries for our system are listed below (chapter 2.1, 0 and 2.3.).

## *1.4 Python usage without an interpreter*

To change the Python code without an interpreter (e.g. Eclipse, Visual studio), go into the “venvXY\Scripts” folder and click on the “Python.exe” file. In the Python.exe write: “import idlelib.idle” and press ENTER to open the Python IDLE. There you can open and manipulate existing Python scripts or write your own. We still recommend to use an interpreter like Visual studio or Eclipse. To change file paths use “/" or “\\” or r’path\to\something’ to define a folder location. Save the changed Python program afterwards. To test the Python program in the IDLE press F5.

Python scripts can be executed over the Windows command line, which is done automatically with our detection system. For manual script execution do the following:

1. Activate the virtual environment
    a. Open the Windows command window (Windows + R → cmd)
    b. Write your virtual environment activate path e.g.: “venv38\Scripts\activate” and press ENTER
    c. Write “cd C:\Users\name\camera_detection_system\” and press ENTER. Now, the Windows path changed to the detection system folder.
    d. Start the Python program by typing “3D_reconstruction.py” and press ENTER.

## *1.5 FreeCAD installation*

Download the FreeCAD installer from the FreeCAD official website. Choose either the executable installer or the portable ZIP file. After installation, update the “FreeCAD_path.txt” file in the “camera_detection_system\3D_reconstruction\” directory with your FreeCAD installation path.

### *1.6 User-specific path alterations*

Some paths in the detection system are user-specific. Update the following files in the camera_detection_system folder:

- “display_detection\detection_requirements.txt”: Insert your “Cutout.pro” user name and password as shown in Table S1.
- “3D_reconstruction\FreeCad_path.txt”: Update the FreeCAD bin folder path (“C:\\Users\\users\\AppData\\Local\\Programs\\FreeCAD_0_21\\bin”).

| **Text file line** | **User interaction** |
|---|---|
| 1 | Cutout.pro e-mail |
| 2 | Cutout.pro password |

*Table S1:* Display detection system requirements text file. To use Cutout.pro, the user must define their password and user name.

## 2 Python software and package versions

In this section we provide detailed information about Python versions and libraries required for each subsystem.

### 2.1 Display detection with Python 3.11.2

Install the libraries listed in Table S2 for display detection. The system only works with the listed library versions.

| ***Library name*** | ***Version*** | ***Command*** |
|---|---|---|
| *NumPy* | 1.24.2 | py –m pip install numpy == 1.24.2 |
| *OpenCV contrib* | 4.6.0.66 | py -m pip install opencv-contrib-python==4.6.0.66 |
| *OpenCV* | 4.6.0.66 | Py -m pip install opencv-python == 4.6.0.66 |
| *Imutils* | 0.5.4 | py –m pip install imutils == 0.5.4 |
| *UUID* | 1.30 | py –m pip install uuid == 1.30 |
| *Ultralytics* | 8.1.8 | py –m pip install Ultralytics == 8.1.8 |
| *PyTorch* | Torch vision: 0.16.0<br>Torch: 2.1.0 | py –m pip install torch ==2.1.0<br>py -m pip install torchvision == 0.16.0 |
| *Pandas* | 2.0.1 | py -m pip install pandas == 2.0.1 |
| *Dill* | 0.3.8 | py -m pip install dill == 0.3.8 |
| *OpenPyXL* | 3.1.5 | py -m pip install openpyxl == 3.1.5 |
| *Selenium* | 4.26.1 | py -m pip install selenium == 4.26.1 |
| *Webdriver manager* | 4.0.2 | py -m pip install webdriver-manager == 4.0.2 |
| *PyAutoGUI* | 0.9.54 | py -m pip install pyautogui |

*Table S2: Required Python libraries for Python 3.11.2 display detection.*

## 2.2 3D reconstruction with Python 3.8.1

Using FreeCAD in Python, requires the adaption of the FreeCAD bin folder path in the "FreeCad_path.txt" file. Install the libraries listed in Table S3 for 3D reconstruction.

| ***Library name*** | ***Version*** | ***Command*** |
|---|---|---|
| *NumPy* | 1.24.2 | py –m pip install numpy == 1.24.2 |
| *OpenCV contrib* | 4.6.0.66 | py -m pip install opencv-contrib-python==4.6.0.66 |
| *OpenCV* | 4.6.0.66 | Py -m pip install opencv-python == 4.6.0.66 |
| *Pandas* | 2.0.1 | py -m pip install pandas == 2.0.1 |
| *Scipy* | 1.10.1 | py -m pip install scipy == 1.10.1 |
| *PySide2* | 5.15.2.1 | py -m pip install PySide2 == 5.15.2.1 |
| *Wheel* | 0.41.2 | py -m pip install wheel == 0.41.2 |

*Table S3: Required Python libraries for Python 3.8.1 3D reconstruction.*

## 2.3 Camera calibration with Python 3.12.0

Use the commands in Table S4 to install the required libraries.

| ***Library name*** | ***Version*** | ***Command*** |
|---|---|---|
| *NumPy* | 1.26.2 | py –m pip install numpy == 1.26.2 |
| *OpenCV* | 4.8.1.78 | py -m pip install opencv-contrib-python ==4.8.1.78 |
| *UUID* | 1.30 | py –m pip install uuid == 1.30 |

*Table S4: Required Python libraries for Python 3.12.0 camera calibration*

***Supplementary Information S2 for the Article:***

# Facilitating Laboratory Automation Using A Robot With A Simple And Inexpensive Camera Detection System

Rebecca Wienbruch[a] wienbruch@hs-albsig.de ORCID: 0009-0006-6649-9280, Nicole Rupp[a] rupp@hs-albsig.de ORCID: 0000-0002-7967-1346, Ruven Dreischke[b,1] ruven@dreischke.de, Verena Mayer[a] mayer_verena99@web.de ORCID: 0009-0003-6089-9134, Isabell Fenske[a] isabell.fenske@gmx.de, Rebecca Bauer[a] bauerreb@web.de ORCID: 0009-0000-0116-6908, Thole Zuchner[a,*] ORCID: 0000-0001-6095-4656

a: Faculty for Life Sciences, Professorship for Bioanalytics and Laboratory automation, Albstadt-Sigmaringen University, Anton-Günther-Str. 51, D-72488 Sigmaringen, Germany; b: jetzt GmbH, Schiffstr. 46, D-78467 Konstanz, Germany; [1]Am Speyerbach 44, D-67433 Neustadt Weinstraße, Germany

*<u>*Corresponding author:*</u>
Prof. Dr. Thole Zuchner, Email: zuechner@hs-albsig.de, phone: +49 (0)7571 732 8240

## 1 *Additional results details*

To provide a deeper understanding of our system's outcomes, we present more detailed explanations in the following sections.

### 1.1 *3D-reconstruction - triangulation process*

To extract depth information from 2D images, a triangulation process is used, which relies on pre-processed camera projection matrices and pixel coordinates of ArUco markers. During triangulation, these projection matrices and pixel coordinates are used to identify common points between the two camera perspectives in a local camera coordinate system. This process generates homogeneous 4D coordinate points within the local camera coordinate system. Homogeneous coordinates extend 3D coordinates into a 4D system by appending a fourth line (containing four “1”) to the matrix. Each camera’s position is defined by a translation and a rotation vector, and during triangulation, these transformations are combined. To achieve linear transformation, both the 3D rotation and translation vectors are converted into homogeneous coordinates. [1]

Before triangulation, it is necessary to align the camera and pixel coordinate systems. This is achieved by applying a +90° rotation to the z-coordinate of the rotation vector. The first camera’s position is set as the new origin (translation vector set to zero) to establish a local camera coordinate system. The second camera's position is recalculated relative to the first camera’s position (new origin), adjusting both the rotation and translation vectors. These transformations are used to construct the projection matrices needed for 3D reconstruction. A 3x4 projection matrix transforms points from the 3D camera

coordinate system into 2D image points by applying matrix multiplication between the intrinsic camera matrix and the adjusted extrinsic camera's translation and rotation vectors. [2,3]

To enhance reconstruction accuracy, OpenCV functions such as "undistortPoints" are used to correct distortions caused by the lens's shape and assembly. The camera lens increases the field of view but influences the way of light propagation depending on the lens shape. Straight lines tend to become a curve through the lens, and the closer to the edge of the image/lens, the bigger the distortion. The shape of the lens induces radial distortion that influences the pixel radius. Another source of distortion is the mechanical assembly, which causes the lens and imaging plane not to be entirely parallel. The distortion caused by the assembly is called tangential distortion and influences the projected position. [1]

After distortion correction and projection matrix construction, triangulation is performed using the pre-processed pixel coordinates and projection matrices. This step computes the 3D positions of the ArUco markers as homogeneous 4D coordinate points in the local camera coordinate system. The back-transformation of the 4D homogeneous coordinates into 3D Cartesian robot world coordinates occurs in two steps. First, the 4x4 homogeneous coordinate matrix is reduced to a 3x3 matrix to return to Cartesian coordinates. The fourth row of the homogeneous coordinate matrix represents the homogeneous vector used for the transformation from 4D to 3D. The first three-row elements of the 4x4 matrix are divided by the row's fourth element to transform the coordinates back into the Cartesian coordinate system. The resulting 3D matrix is reshaped afterwards. [4]

At this stage, the Cartesian coordinates remain in the local camera coordinate system. To convert them back into the robot's world coordinate system, the x and y values must be negated. This adjustment accounts for the 90° rotation of the original coordinates into the local camera coordinate system, which caused the x and y axes of the pixel and camera coordinate systems to align in opposing directions. Additionally, the initial translation vector of the first camera (original position) is added to the coordinate points to correct for the shift in the coordinate systems' origin. Finally, the coordinates are transformed back into the robot's world coordinate system, making them ready for further calculations.

## *1.2 Display detection - trained model development*

During image training, YOLOv8 evaluates various parameters, such as the quality of recognized bounding boxes, to assess the success of each training cycle. To determine whether a prediction is positive (correct) or negative (false), a threshold value is required. One such threshold is the IoU

(Intersection over Union), which expresses the overlap between the predicted bounding box and the ground truth box. A higher IoU threshold increases the occurrence of false negatives, whereas a lower IoU threshold increases the occurrence of false positives. Therefore, the threshold value must be carefully considered as it directly influences the quality of the training model.

The mAP50 (mean average precision for a threshold of 50 % bounding box overlap) parameter determines the model's average precision by considering a range of predictions. Specifically, mAP50 filters predictions with an IoU value greater than 50 % and calculates the average precision across all classes. In simpler terms, mAP50 measures the model´s accuracy for "easy" detections, as the Ultralytics website claims [5]. The mAP50 values of our trained models are shown in Fig. S1. Model "V9" achieved the highest mAP50 value (mAP50 = 0.969), while model "V10" achieved the best predictions results (mAP50 = 0.956), as this model was trained without images of the "Thermo Scientific Shaking Drybath".

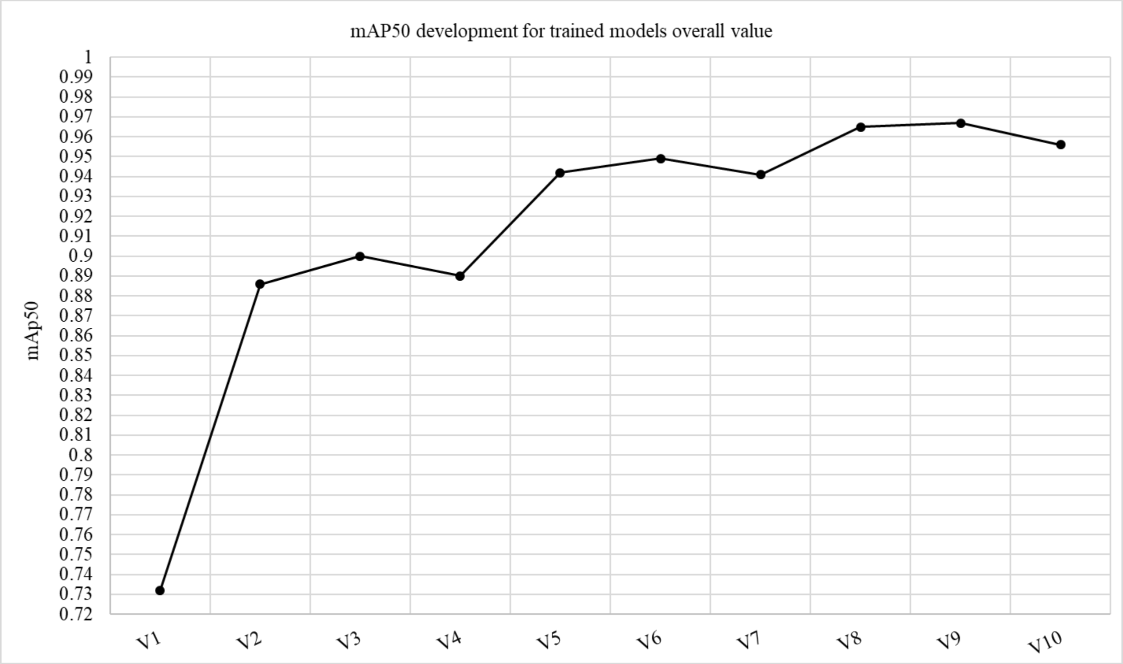


**Fig. S1** Trained model's mean average precision (mAP) for a threshold of 50 % bounding box overlap. The higher the mAP50, the higher the model's accuracy. Model "V9" achieved the highest mAP50 value, while model "V10" achieved the best prediction results.

| *Class Name* | *Total Count* | *Training Count* | *Validation Count* |
|---|---|---|---|
| *.* | 654 | 539 | 115 |
| *1* | 494 | 418 | 76 |
| *2* | 471 | 402 | 69 |
| *0* | 367 | 303 | 64 |
| *3* | 334 | 319 | 15 |
| *pH* | 309 | 207 | 102 |
| *9* | 304 | 272 | 32 |
| *5* | 284 | 268 | 16 |
| *6* | 264 | 237 | 27 |
| *8* | 259 | 240 | 19 |
| *g* | 250 | 250 | 0 |
| *4* | 243 | 220 | 23 |
| *7* | 243 | 224 | 19 |
| *°C* | 158 | 107 | 51 |
| *lid* | 153 | 143 | 10 |
| *min* | 75 | 64 | 11 |
| *rpm* | 43 | 36 | 7 |
| *OFF* | 10 | 10 | 0 |

**Table S1** Class distribution for display detection model V10. The total class distribution is compared to the training and validation subset distributions. The training subset has a more homogenic class distribution than the validation subset.

Table S2 represents the detailed display detection results for the Tesseract OCR system. For this testing a Python 3.12 script with pytesseract version 0.3.13.

| | ***FCE 3K1N*** | ***Micro Star 12*** | ***CS200*** | ***RSM-10HP*** | ***pH 50+ DHS*** |
|---|---|---|---|---|---|
| *Manufacturer* | KERN & SOHN GmbH | VWR International | OHAUS Europe GmbH | Phoenix Instrument GmbH | XS Instruments |
| Country | Germany | USA | Switzerland | Germany | Italy |
| *Font* | Black | Light blue | Black | Red | Black |
| *Background* | Grey | Dark blue | Light grey | Dark blue | Light blue |
| *Illumination* | No | Yes | No | Yes | Yes |
| *Value 1 limits* | 0 - 3000 | 1.2 - 13.5 | 0.0 - 200.0 | 5 - 280 | 0 - 14 |
| *Value 2 limits* | - | 1 - 30 | - | 200 - 1500 | 0 - 100.0 |
| *Value 1 decimal places* | None | 1 | 1 | None | 2 |
| *Value 2 decimal places* | - | None | - | None | 1 |
| *Tested images* | 10 | 10 | 10 | 10 | 10 |
| *Value 1 correct* | 0% | 0.00% | 0% | 0% | 0% |
| *Value 2 correct* | - | 10% | - | 10% | 0% |
| *Sum detectable digits* | 19 | 44 | 40 | 54 | 80 |
| *Sum correct digits* | 1 | 0 | 10 | 10 | 23 |
| *Sum false digits* | 10 | 1 | 11 | 1 | 15 |
| *Sum missing digits* | 8 | 43 | 21 | 43 | 42 |
| *Sum additional digits* | 0 | 0 | 2 | 0 | 0 |
| *Sum detected digits* | 11.00 | 1.00 | 21.00 | 11.00 | 38.00 |
| *Error rate* | 0.95 | 1.00 | 0.85 | 0.81 | 0.71 |
| *Recall* | 0.05 | 0.00 | 0.25 | 0.19 | 0.29 |
| *Precision* | 0.09 | 0.00 | 0.48 | 0.91 | 0.61 |

**Table S2** Tesseract OCR display detection results. In total 50 images, with 237 detectable digits were used.

## 2 References

1. Gao, X. & Zhang, T. *Introduction to Visual SLAM* (Springer Singapore, 2021).
2. Zhang, Z. in *Computer Vision,* edited by K. Ikeuchi (Springer International Publishing, 2021), pp. 130–131.
3. Zhang, Z. in *Computer Vision,* edited by K. Ikeuchi (Springer International Publishing, 2021), pp. 520–526.
4. OpenCV. Camera Calibration and 3D Reconstruction. Available at https://docs.opencv.org/3.4/d9/d0c/group__calib3d.html (2023).
5. Jocher, G., Waxmann, S. & Chaurasia, A. Ultralytics YOLOv8. Available at https://docs.ultralytics.com (2022).
